# Contribution of oxidative stress and growth factor receptor transactivation in natriuretic peptide receptor C-mediated attenuation of hyperproliferation of vascular smooth muscle cells from SHR

**DOI:** 10.1371/journal.pone.0191743

**Published:** 2018-01-24

**Authors:** Sofiane Rahali, Yuan Li, Madhu B. Anand-Srivastava

**Affiliations:** Department of Pharmacology and Physiology, Faculty of Medicine, University of Montreal, Quebec, Canada; Qatar University College of Health Sciences, QATAR

## Abstract

Earlier studies have shown the implication of growth factor receptor activation in angiotensin II (Ang II)-induced hyperproliferation of aortic VSMC as well as in hyperproliferation of VSMC from spontaneously hypertensive rats (SHR). We previously showed that NPR-C specific agonist C-ANP_4-23_ attenuates the hyperproliferation of VSMC from SHR through the inhibition of MAP kinase, Giα protein signaling and overexpression of cell cycle proteins. The aim of the present study was to investigate if C-ANP_4-23_- mediated attenuation of hyperproliferation of VSMC from SHR also involves growth factor receptor activation and upstream signaling molecules. For this study, C-ANP _4–23_ (10 nmole/kg body weight) was injected intraperitoneally into 2 week-old prehypertensive SHR and Wistar Kyoto (WKY) rats twice per week for 6 weeks. The blood pressure in SHR was significantly attenuated by C-ANP_4-23_ treatment. In addition, C-ANP_4-23_ treatment also attenuated the hyperproliferation of VSMC from SHR as well as the enhanced phosphorylation of EGF-R, PDGF-R, IGF-R and c-Src. Furthermore, the enhanced levels of superoxide anion, NADPH oxidase activity, and enhanced expression of Nox4,Nox1,Nox2 and P47^phox^ in SHR compared to WKY rats was also significantly attenuated by C-ANP_4-23_ treatment. In addition, N-acetyl cysteine (NAC), a scavenger of O_2_^-^, inhibitors of growth factor receptors and of c-Src, all inhibited the overexpression of cell cycle proteins cyclin D1 and cdk4 in VSMC from SHR. These results suggest that in vivo treatment of SHR with C-ANP_4-23_ inhibits the enhanced oxidative stress, c-Src and EGF-R, PDGF-R, IGF-R activation which through the inhibition of overexpression of cell cycle proteins result in the attenuation of hyperproliferation of VSMC.

## 1. Introduction

Atrial natriuretic peptide (ANP), brain natriuretic peptide (BNP) and C-type natriuretic peptide (CNP) belong to a family of natriuretic peptides (NP)[1, 2] and regulate physiological functions through their interaction with their receptors NPR-A, NPR-B and NPR-C[3]. NPR-A and NPR-B are membrane guanylyl cyclase receptors whereas NPR-C is coupled to adenylyl cyclase inhibition through inhibitory guanine nucleotide regulatory protein Gi [4, 5] or to activation of phospholipase C [6]. However, we showed that NPR-C-mediated decrease in cAMP levels contribute to the activation of PLC signaling and suggested a cross talk between NPR-C-mediated adenylyl cyclase and PLC signaling pathways [7].

Hyperproliferation of vascular smooth muscle cell (VSMC) due to a phenotypic change contributes to vascular remodelling and is considered as one of the major cellular events involved in many VSMC-related pathological conditions, such as atherosclerosis, diabetes and hypertension [8–11]. The enhanced proliferation of VSMC from spontaneously hypertensive rats (SHR) has been reported by several studies [12, 13]. Bou Daou et.al [14] have recently shown the implication of enhanced expression of Giα proteins in hyperproliferation of VSMC from SHR. In addition, the enhanced levels of endogenous vasoactive peptides including angiotensin II (ANG II) and endothelin-1 (ET-1) exhibited by VSMC from SHR [15, 16] were also shown to contribute to the hyperproliferation through oxidative stress, transactivation of epidermal growth factor receptor (EGF-R) and MAP kinase signaling pathways [11].

C-ANP_4-23_, a specific agonist that interacts with NPR-C as well as the short cytoplasmic domain peptide of NPR-C containing G_i_ activator sequence have been shown to attenuate the hyperproliferation of VSMC induced by growth factors and vasoactive peptides [17, 18]. We recently showed that C-ANP_4-23_ also inhibited the hyperproliferation of aortic VSMC from SHR and demonstrated the contribution of cell cycle proteins/ cyclin-dependent kinases (CDK) as well as Giα protein and MAP kinase signaling in C-ANP_4-23_-mediated inhibition of hyperproliferation of VSMC from SHR [19]. However, the implication of growth factor receptor transactivation and upstream signaling molecules in NPR-C-mediated attenuation of hyperproliferation of VSMC from SHR has not been explored. The present study therefore investigates the contribution of growth factor receptor transactivation, oxidative stress and c-Src in NPR-C-mediated attenuation of hyperproliferation of vascular smooth muscle cells from SHR. We have shown for the first time that in vivo treatment of SHR with C-ANP_4-23_ inhibits the enhanced oxidative stress, c-Src and growth factor receptor activation which through the inhibition of overexpression of cell cycle proteins result in the attenuation of hyperproliferation of VSMC.

## 2. Materials and methods

### 2.1 Materials

C-ANP _4–23_ was purchased from Bachem, antibodies against Cyclin D1, Cdk4, total EGF-R, phospho-EGFR, total PDGF-R β, phospho-PDGF-R β, total IGF-R β, phospho-IGF-IR, c-Src, phosphor-c-Src, horseradish peroxidase-conjugated goat anti-mouse/anti-rabbit and anti-goat immunoglobulin were from Santa Cruz Biotechnology Inc (Santa Cruz, CA, USA), antibodies against Nox1, Nox2, Nox4 and p47^phox^ were from EMD Millipore. Thymidine, [Methyl-3H] was from PerkinElmer, Inc. (Massachusetts, U.S.A.). All other chemicals were purchased from Sigma Aldrich Canada.

### 2.2 Animal treatment

One-week-old-male spontaneously hypertensive rats (SHR) and age-matched normotensive Wistar-Kyoto (WKY) rats were purchased from Charles River Laboratories Canada (St-Constant, Qc, Canada). Animals were maintained at room temperature in 12h light -dark cycles. Rats were left for 1 week for adaptation. SHR and WKY rats were divided into 4 groups (Control WKY and SHR and C-ANP_4-23_-treated WKY and SHR) (6 rats / group). Two week-old SHR and age-matched WKY rats were injected intraperitoneally with C-ANP_4–23_ (10 nmol/kg body weight) twice per week for 6 weeks in 0.01 mol/L sodium phosphate buffer, pH 7.0, containing 0.05 mol/L NaCl as described previously [20]. The control WKY rats and SHR received vehicle. The blood pressure was monitored twice a week by tail-cuff method without anesthesia using CODA standard non-invasive blood pressure system. At the end of the 8th week, after taking the blood pressure, the rats were euthanized by decapitation after CO exposure. The thoracic aorta were dissected out and used for cell culture. All the animal procedures used in the present study were approved by the Comité de Déontologie de l’Expérimentation sur les Animaux (CDEA) of the University of Montreal (protocol #99050). The investigation conforms to the Guide for the Care and Use of Laboratory Animals published by the US National Institutes of Health (Guide, NRC 2011).

### 2.3 Cell culture

Aortic VSMCs from 8-week-old SHRs and WKY rats (control group) and C-ANP_4–23_–treated SHRs and WKY rats were cultured as described in detail previously [17, 19, 21]. As reported earlier [22] these cells were found to contain high levels of smooth-muscle-specific actin. The cells were plated in 75 cm^2^ flasks and incubated at 37°C in 95% air and 5% CO_2_ humidified atmosphere in Dulbecco’s modified Eagle’s medium (DMEM) (with glucose, l-glutamine, and sodium bicarbonate) containing antibiotics and 10% heat-inactivated fetal bovine serum (FBS). The cells were passaged upon reaching confluence with 0.5% trypsin containing 0.2% EDTA and utilized between passages 3 and 10. Confluent cells were then starved by incubation for 24 h in DMEM without FBS at 37°C to reduce the interference by growth factors present in the serum. The cells were then incubated in the absence or presence of N-acetyl cysteine (NAC) (10 mM), PP2, PP3 (5 μM), AG1295 and AG1478 (5 μM) for 24 h. After incubation, the cells were washed three times with PBS and lysed in 30 ml of buffer (25 mM Tris-HCl, pH 7.5, 25 mM NaCl, 1 mM Na orthovanadate, 10 mM Na fluoride, 10 mM Na pyrophosphate, 2 mM ethylene, bis(oxyethylenenitrolo) tetracetic acid, 2 mM ethylenediamine tetracetic acid, 1 mM phenylmethylsulfonyl fluoride, 10 mg/ml aprotinin, 1% Triton X-100, 0.1% sodium dodecyl sulphate (SDS), and 0.5 mg/ml leupeptin) on ice. The cell lysates were centrifuged at 12,000 g for 15 min at 4°C, and the supernatants were used for Western blot analysis. Cell viability was checked by the trypan blue exclusion technique and indicated that >90~95% cells were viable. Protein concentration was determined by Bradford assay [23].

### 2.4 Western blot analysis

The levels of cyclinD1, cdk4 Nox1, Nox2, Nox4, p47^phox^, c-Src and growth factor receptors were determined by Western blotting using specific antibodies as described in detail earlier [19, 24]. Equal amounts of protein (30 μg) were subjected to 10% SDS-polyacrylamide gel electrophoresis (SDS-PAGE), transferred to nitrocellulose membranes and incubated with the respective primary antibodies: cyclin D1, Cdk4, c-Src, phospho-c-Src, EGF-R, phospho-EGFR, PDGF-R β, phospho-PDGF-R β, IGF-R β, phospho-IGF-IR, Nox1, Nox2, Nox4 and phosphor-p47^phox^. The antibody-antigen complexes were detected by second antibody horseradish peroxidase-conjugated goat anti-mouse, donkey anti-goat and goat anti-mouse. Protein bands were visualized by enhanced-chemiluminescence(ECL). Western blotting detection reagents were from Santa Cruz Biotechnology. Quantitative analysis of specific bands was performed by densitometric scanning of the autoradiographs with an enhanced laser densitometer (LKB Ultroscan XL, Pharmacia, Dorval, Qc, Canada) and quantified by using gel-scan XL evaluation software (version 2.1) from Pharmacia.

### 2.5 Determination of superoxide anion production and NADPH oxidase activity

Basal superoxide anion production and NADPH oxidase activity in the VSMCs were measured using the lucigenin-enhanced chemiluminescence method with low concentration (5 μmol/l) of lucigenin as described previously [24]. VSMC from control and C-ANP _4-23_-treated SHR and WKY rats were washed in oxygenated Kreb–Hepes buffer, and placed in scintillation vials containing lucigenin solution, and the emitted luminescence was measured with a liquid scintillation counter (Wallace 1409: Turku, Finland) for 5 min. The average luminescence value was estimated, the background value subtracted and the result was divided by the total wet weight of tissue in each sample. The NADPH oxidase activity in the samples was assessed by adding 10^-4^mol/l NADH (Sigma Chemical Co.) in the vials before counting. Basal superoxide- induced luminescence was then subtracted from the luminescence value induced by NADH.

### 2.6 [Methyl-3H] thymidine incorporation

DNA synthesis was evaluated by incorporation of [^3^H] thymidine into cells as described in detail earlier [17, 19]. Subconfluent VSMC from control and C-ANP_4-23_ treated SHR and WKY rats were plated in 6-well plates for 24 hrs and were serum deprived for 24 h to induce cell quiescence. [^3^H] thymidine (1 μCi/ml) was added and further incubated for 4 h before the cells were harvested. The cells were rinsed twice with ice-cold PBS and incubated with 5% trichloroacetic acid for 1 h at 4°C. After being washed twice with ice-cold water, the cells were incubated with 0.4 N sodium hydroxide solution for 30 min at room temperature, and radioactivity was determined by liquid scintillation counter. Cell viability was checked by the trypan blue exclusion technique and indicated that >90~95% cells were viable.

### 2.7 Statistical analysis

The number of independent experiments is reported. Each experiment was conducted at least five times using separate cell population. All data are expressed as the mean ± SEM. Comparisons between groups were made with one way analysis of variance (ANOVA) followed by Dunnett tests using GraphPad Prism5 software. Results were considered significant at a value of p < 0.05.

## 3. Results

The mean blood pressure (BP) of SHR and WKY rats at 8 weeks were 191 ± 3.5 mmHg and 108 ± 8.1 mmHg respectively. Intraperitoneal injection of C-ANP_4–23_ (10 nmol/kg BW) for six weeks (twice weekly) decreased the BP in SHR by 72 mmHg (118 ± 9.8 mmHg vs 191 ± 3.5 mmHg) without affecting the BP in WKY rats. In addition, C-ANP_4–23_ did not have any adverse effects on the health of the animals, because all rats treated with C-ANP_4–23_ maintained or gained weight during the period of the studies (Body weight (g) WKY CTL 162±5.8 WKY+ C-ANP_4-23_ 164±6.1 SHR CTL 153±8.1 SHR +C-ANP_4-23_ 151±5).

### 3.1 In vivo treatment of C-ANP_4–23_ attenuates hyperproliferation of VSMC from SHR

**Fig 1:**
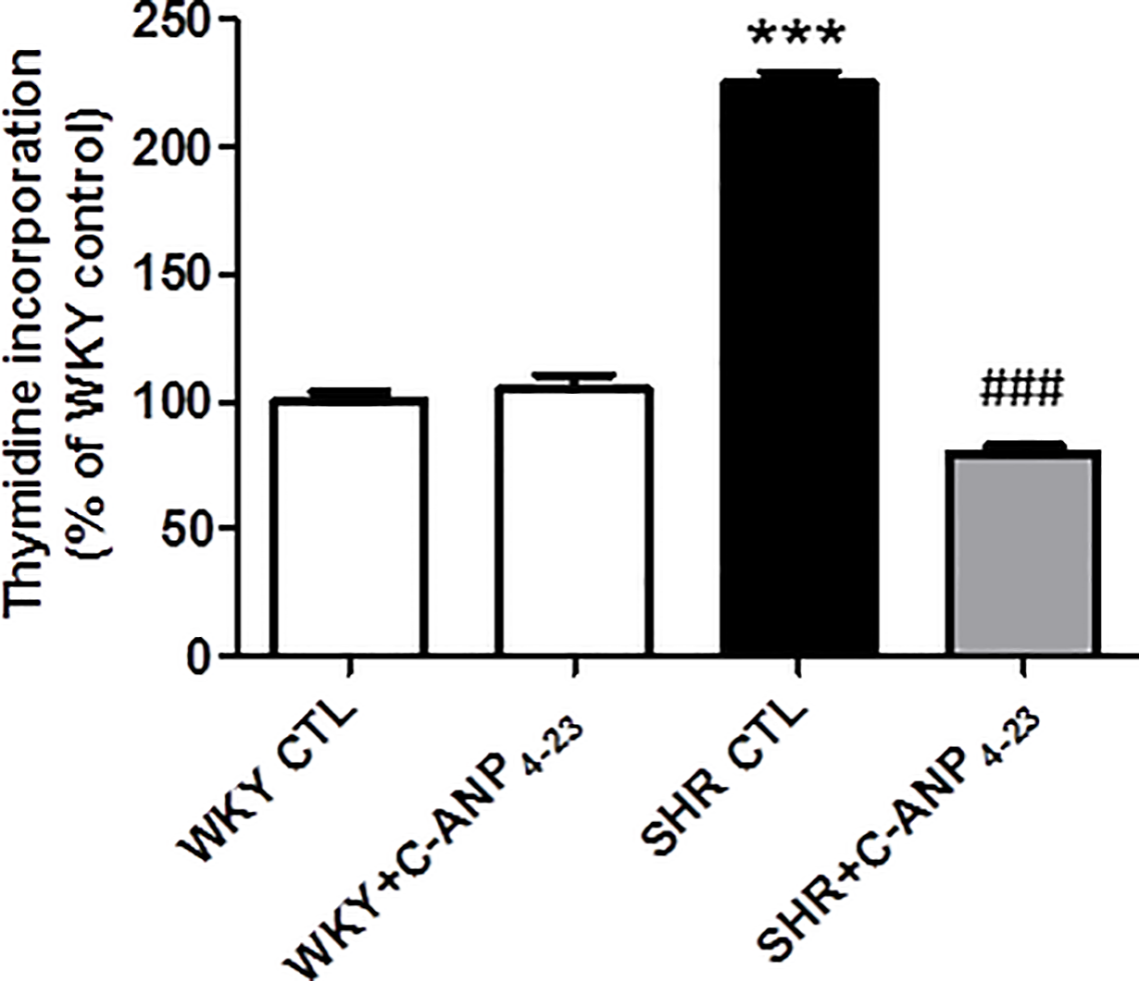
Effect of *in vivo* C-ANP _4–23_ treatment on DNA synthesis in Vascular Smooth Muscle Cells (VSMC) from 9 week-old SHR and age-matched WKY. One week old SHR and age matched WKY rats (control) were injected intraperitoneally with C-ANP_4–23_ (10 nmol/Kg of body weight) twice weekly up to 8 weeks as described in ‘‘Materials and Methods”. After eight weeks of treatment, the rats (9 week-old) were sacrificed and aortic VSMC from SHR and age-matched WKY (control groups) and C-ANP _4–23_ treated groups were cultured and thymidine incorporation was determined as described in ‘‘Materials and Methods”. Results are expressed as % of WKY CTL, taken as 100%. Values are means ± SEM of 6 separate experiments using different cell populations. ***P<0.001 vs WKY CTL, ^###^P<0.001 vs SHR CTL.

“Fig 1” shows the effect of in vivo treatment of C-ANP_4-23_ on proliferation of VSMC from SHR and WKY rats. VSMC from SHR exhibited enhanced proliferation as compared WKY rats by about 125% as determined by thymidine incorporation and this enhanced proliferation was significantly restored to control levels by C-ANP_4-23_ treatment. On the other hand, C-ANP_4-23_ treatment did not have any significant effect on the proliferation of VSMC from WKY rats.

### 3.2 In vivo C-ANP_4-23_ treatment attenuates the enhanced phosphorylation of c-Src and growth factor receptors in VSMC from SHR

**Fig 2:**
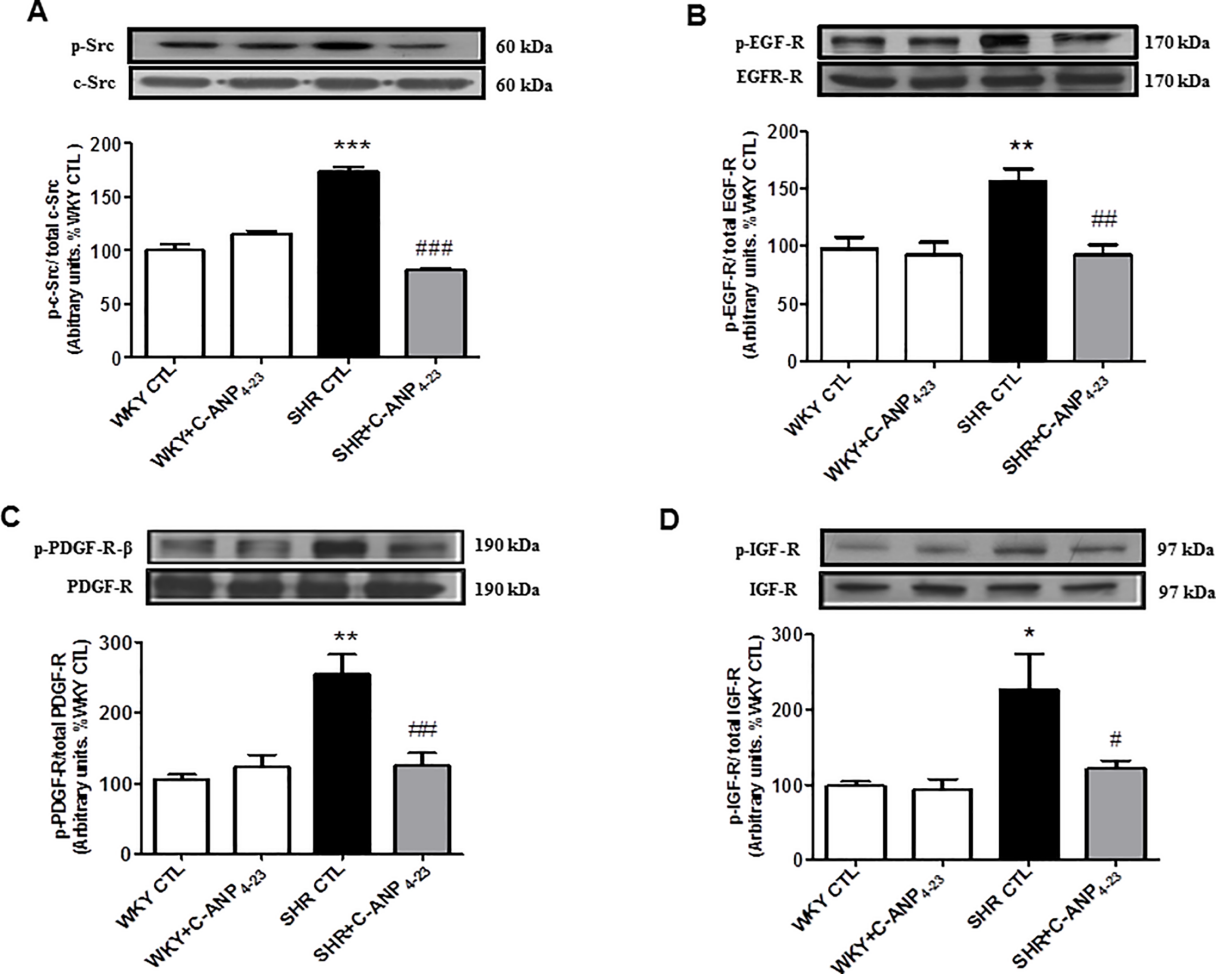
Effect of in vivo C-ANP4-23 treatment on the enhanced phosphorylation of c-Src and growth factor receptors in VSMC from 9 week-old SHRs and age-matched WKY rats. VSMC lysates from from 9-week-old SHR and WKY rats with or without C-ANP4–23 treatment were subjected to Western blotting using specific antibodies against phospho-c-Src/c-Src (A), phospho-EGF-R/EGF-R (B), phospho-PDGF-R/PDGF-R (C) and phospho-IGF-R/IGF-R (D) as described in ‘‘Materials and Methods”. The proteins were quantified by densitometric scanning as described in materials and methods. Results are expressed as % of WKY CTL, taken as 100%.Values are means ± SEM of 6 separate experiments using different cell populations. *P<0.05,**P<0.01, ***P<0.001 vs WKY CTL, ^#^P<0.05, ^##^P<0.01 ^###^P<0.001 vs SHR CTL.

We previously showed the implication of non-receptor tyrosine kinase c-Src in the hyperproliferation of VSMC from SHR [11]. To investigate if C-ANP_4-23_-induced antiproliferative effect is mediated through its ability to decrease the activation of c-Src, the effect of in vivo C-ANP_4-23_ treatment on the phosphorylation of c-Src and growth factor receptors was determined in VSMC from SHR and WKY rats and the results are shown in “Fig 2”. As reported earlier [25], the phosphorylation of Tyr^418^ on c-Src (A) was significantly augmented by about 75% in VSMC from SHR compared with WKY rats and *in vivo* C-ANP_4-23_ treatment completely abolished the enhanced phosphorylation of c-Src in VSMC from SHR. On the other hand, this treatment did not have any significant effect on the phosphorylation of c-Src in WKY rats.

Since the enhanced activation of growth factor receptors reported in VSMC from SHR [22] was shown to contribute to increased proliferation of VSMC from SHR [11], it was of interest to investigate if the antiproliferative effect of in vivo C-ANP _4–23_ treatment is also attributed to its ability to attenuate the enhanced activation of growth factor receptors. To examine this, the effect of in vivo C-ANP_4-23_ treatment on the phosphorylation of EGF-R, PDGF-R and IGF-R was investigated and the results are shown in “Fig 2”. The levels of phosphorylated EGF-R (B) PDGF-R (C) and IGF-R (D) were increased by about 55%, 155% and 125% respectively and that in vivo C-ANP _4–23_ treatment attenuated the enhanced phosphorylation of PDGF-R and IGF-R by about 85% whereas the enhanced phosphorylation of EGF-R was restored to WKY control levels. On the other hand, the basal phosphorylation of these growth factor receptors was not affected by C-ANP_4-23_ treatment in VSMC from WKY rats.

### 3.3 In vivo C-ANP_4–23_ treatment attenuates the enhanced production of superoxide anion, NADPH oxidase activity and enhanced expression of NADPH oxidase subunits in VSMC from SHR

**Fig 3:**
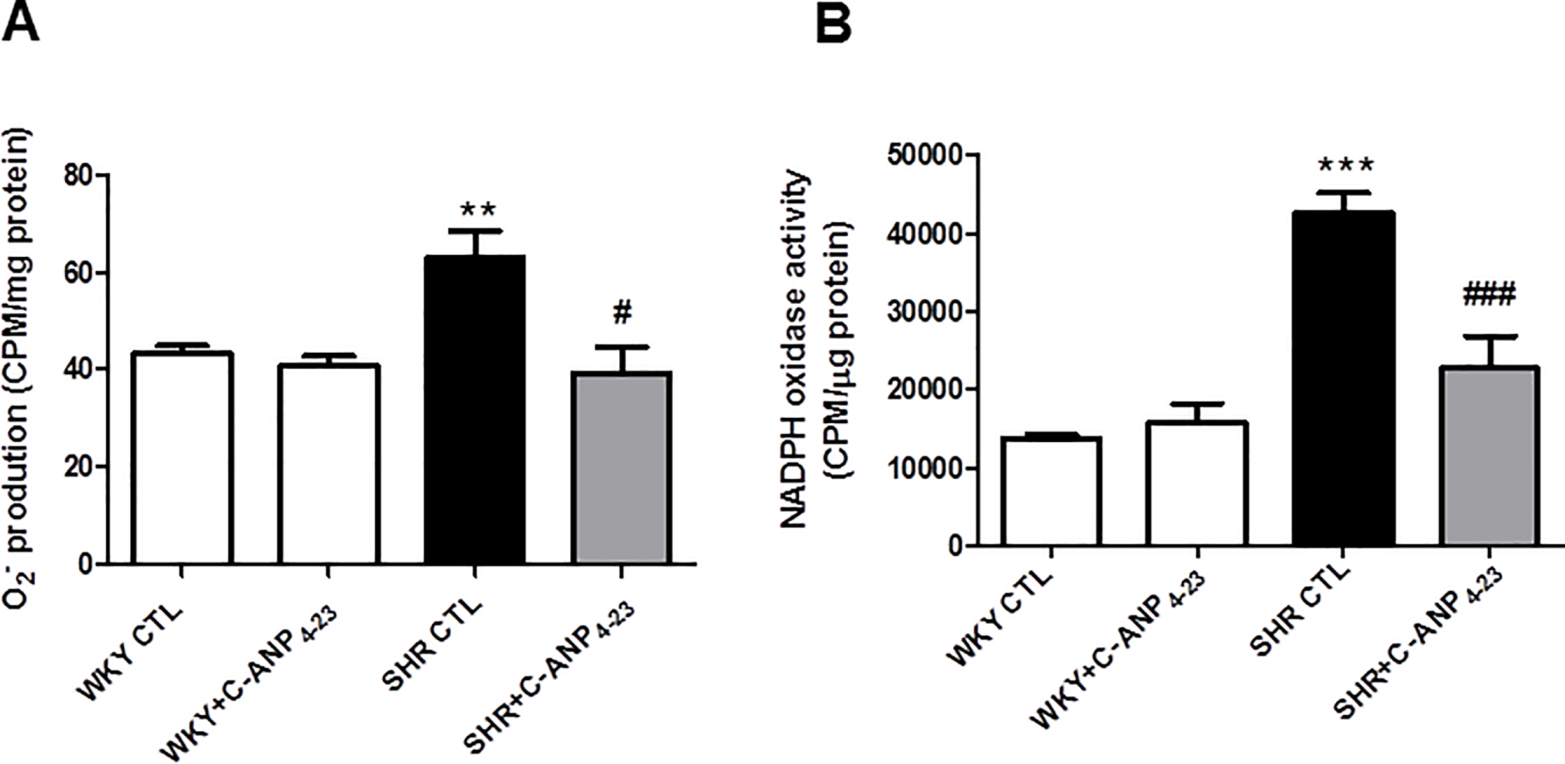
Effect of *in vivo* C-ANP _4–23_ treatment on O_2_^-^ production and NADPH oxidase activity in Vascular Smooth Muscle Cells (VSMC) from 9 week-old SHR and age-matched WKY rats. O_2_^−^ production (A) and NADPH oxidase activity (B) were determined in aortic VSMC from 9-week-old SHR and WKY with or without C-ANP _4–23_ treatment as described in ‘‘Materials and Methods”. Values are means ± SEM of 6 separate experiments using different cell populations. **P<0.01, ***P<0.001 vs WKY CTL, ^#^P<0.05, ^###^P<0.001 vs SHR CTL.

**Fig 4:**
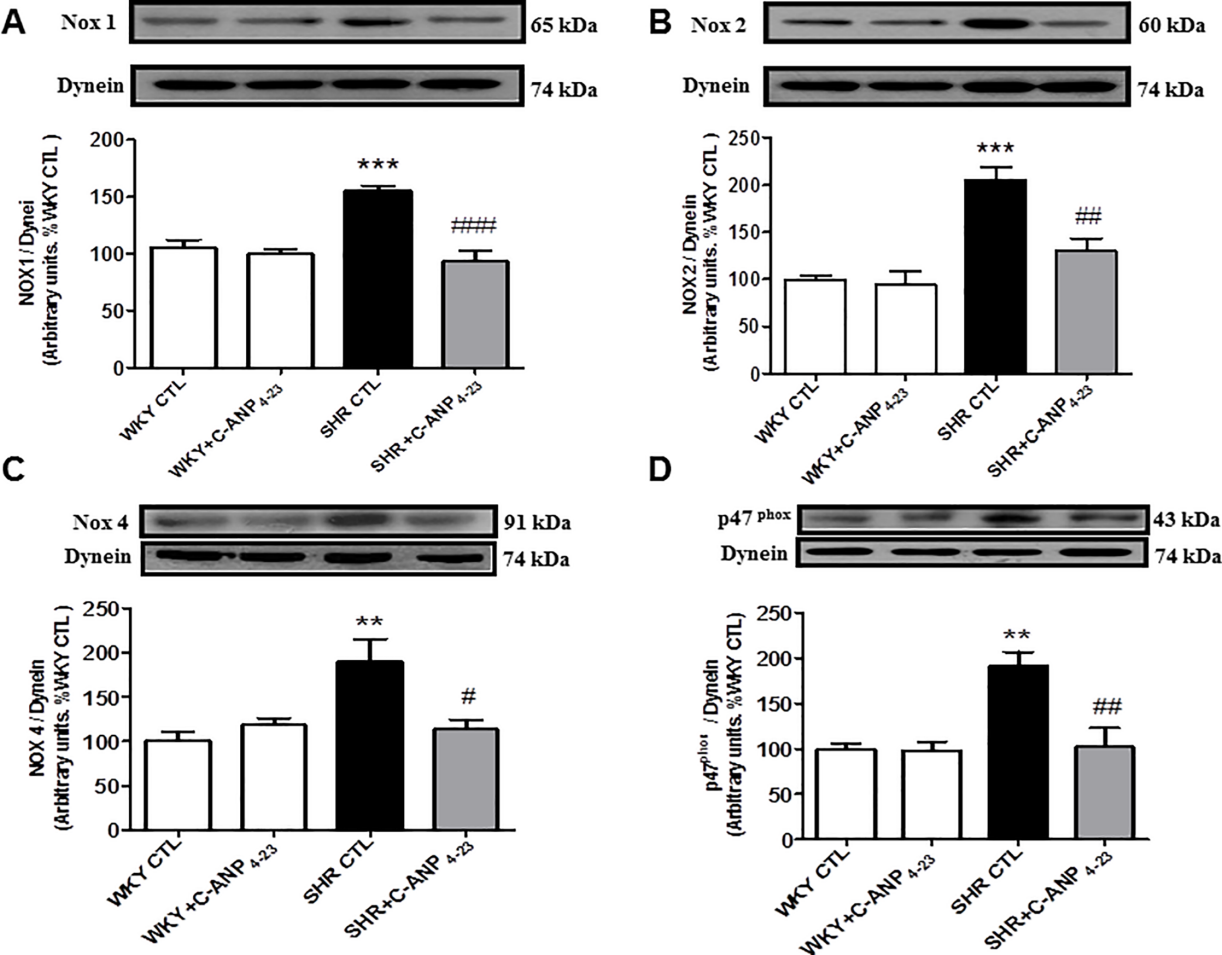
Effect of *in vivo* C-ANP_4-23_ treatment on the expression of subunits of NADPH oxidase in Vascular Smooth Muscle Cells (VSMC) from 9 week-old SHR and and age-matched WKY rats. VSMC lysates from from 9-week-old SHR and WKY rats with or without C-ANP_4–23_ treatment were subjected to Western blotting using specific antibodies against Nox1 (A), Nox2 (B), Nox4 (C) and P47^phox^ (D). Dynein was used as a loading control. The proteins were quantified by densitometric scanning as described in ‘‘Materials and Methods”. Results are expressed as % of WKY CTL, taken as 100%. Values are means ± SEM of 6 separate experiments using different cell populations. **P<0.01, ***P<0.001 vs WKY CTL, ^#^P<0.05, ^##^P<0.01 ^###^P<0.001 vs SHR CTL.

Since oxidative stress has been shown to contribute to hyperproliferation of VSMC from SHR [11] and C-ANP_4-23_ attenuates hyperproliferation, therefore, it was of interest to investigate if the antiproliferative effect of C-ANP_4-23_ is attributed to its ability to decrease enhanced oxidative stress. To test this, the effect of in vivo treatment of C-ANP_4-23_ on the levels of O_2_^-^, NADPH oxidase activity was determined in VSMC from SHR and aged-matched WKY rats. Results shown in “Fig 3” indicate that the level of O_2_^-^ (A) and NADPH oxidase activity (B) were significantly augmented by about 50% and 210% respectively in VSMC from SHR as compared to WKY rats and C-ANP_4–23_ treatment restored the enhanced levels of O_2_^-^ anion to WKY control levels and attenuated the enhanced NADPH oxidase activity by about 80%. In addition, the effect of in vivo C-ANP_4–23_ treatment on the expression of subunits of NADPH oxidase were also examined in VSMC from WKY and SHR and the results are illustrated in “Fig 4”. The levels of Nox1 (A) Nox2 (B), Nox4 (C), and p47phox (D) were significantly enhanced by 50%, 90%, 90% and 90% respectively as compared with WKY rats, and this increase was attenuated to WKY control levels by C-ANP_4-23_ treatment. On the other hand, this treatment did not affect the expression of Nox1, Nox2, Nox4 and p47phox in WKY rats.

### 3.4 Role of ROS, c-Src and growth factor receptor in enhanced expression of cell cycle proteins in VSMC from SHR

**Fig 5:**
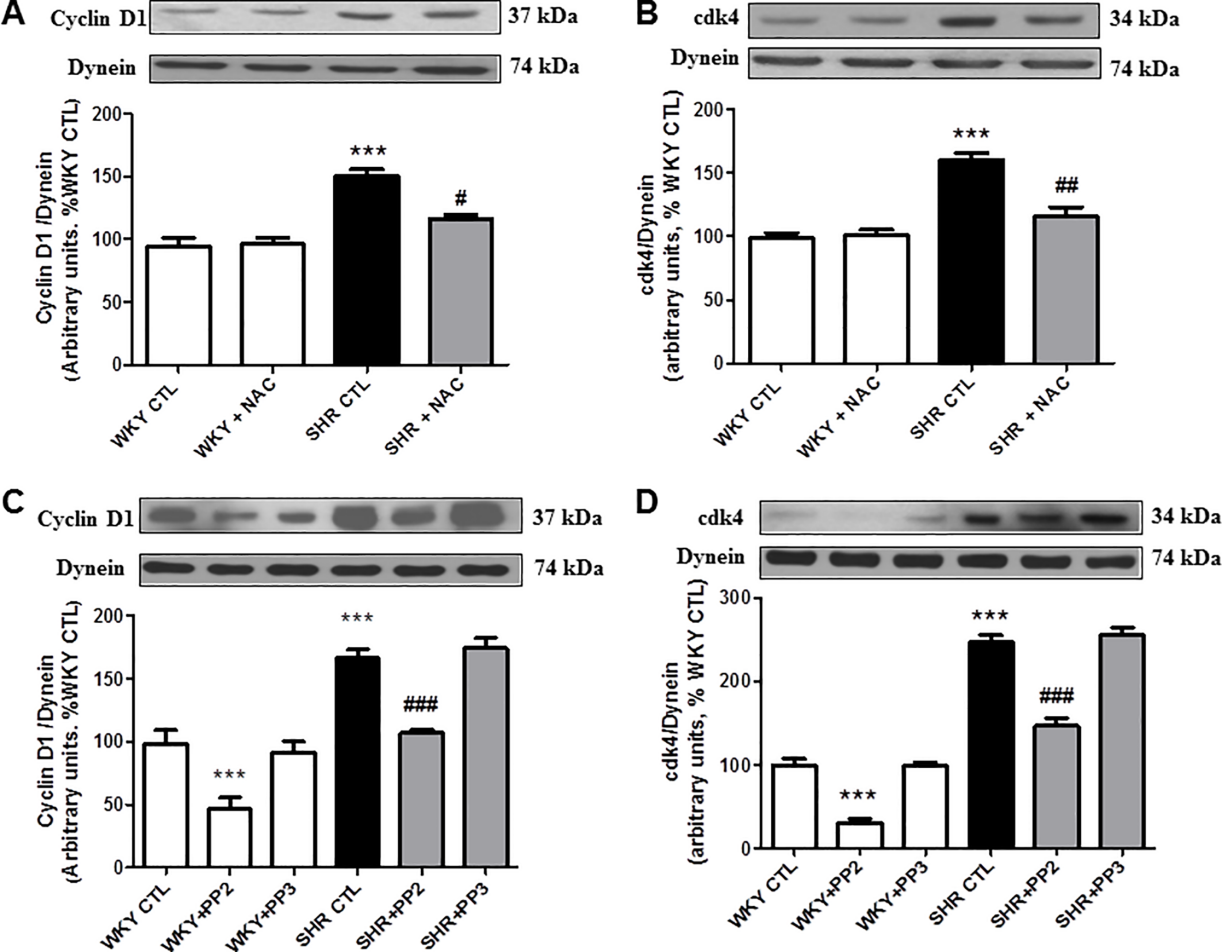
Effect of N-Acetyl-L-cysteine (NAC) and PP2 on the expression of cell cycle proteins in VSMCs from *in vivo* C-ANP_4-23_ treatment SHRs and age-matched WKY rats. Confluent VSMC from 9-week-old SHR and WKY rats with or without C-ANP_4–23_ treatment were starved for 24 h and incubated in the absence (control) or presence of NAC (10 mM) (A and B) or PP2 or PP3 (5 μM) (C and D) for 24 h. The cell lysates were subjected to Western blotting using specific antibodies against cyclin D1 (A and C) and cdk4 (B and D) as described in ‘‘Materials and Methods”. Dynein was used as a loading control. The proteins were quantified by densitometric scanning as described in ‘‘Materials and Methods”. Results are expressed as % of WKY CTL, taken as 100%. Values are means ± SEM of 5 separate experiments using different cell populations. ***P<0.001vs WKY CTL, ^#^P<0.05, ^##^P<0.01, ^###^P<0.001 vs SHR CTL.

**Fig 6:**
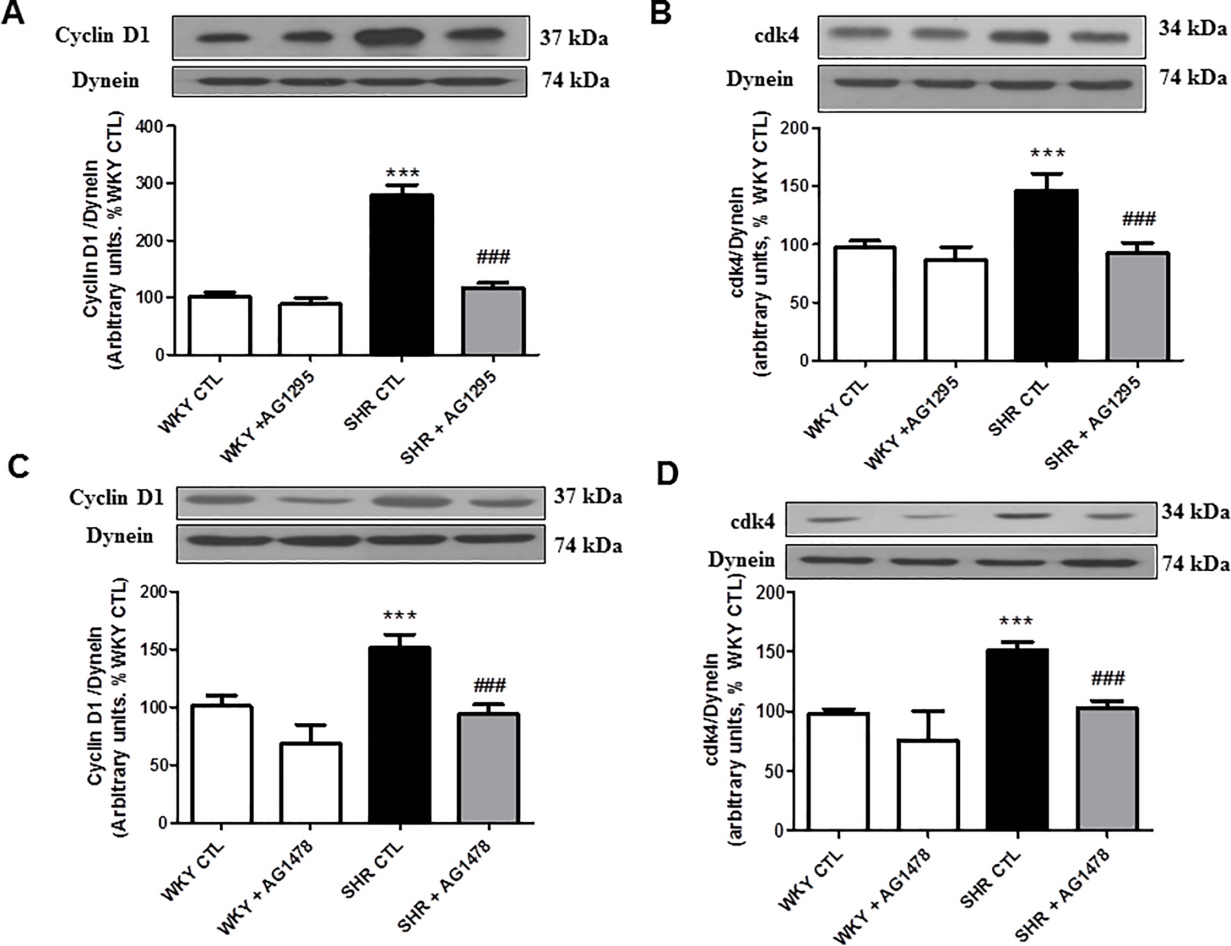
Effect of growth factor receptor inhibitor on the expression of cell cycle proteins in VSMCs from *in vivo* C-ANP_4-23_ treatment SHRs and age-matched WKY rats. Confluent VSMC from 9-week-old SHR and WKY rats with or without C-ANP_4–23_ treatment were starved for 24 h and incubated in the absence (control) or presence of AG1295 (5 μM) (A and B) or AG1478 (5 μM) (C and D) for 24 h. The cell lysates were subjected to Western blotting using specific antibodies against cyclin D1 (A and C) and cdk4 (B and D) as described in ‘‘Materials and Methods”. Dynein was used as a loading control. The proteins were quantified by densitometric scanning as described in ‘‘Materials and Methods”. Results are expressed as % of WKY CTL, taken as 100%. Values are means ± SEM of 5 separate experiments using different cell populations. ***P<0.001vs WKY CTL, ^###^P<0.001 vs SHR CTL.

Since C-ANP_4-23_ inhibits the enhanced oxidative stress, c-Src and growth factor receptor activation, it was of interest to investigate the contribution of these signaling molecules in the overexpression of cell cycle proteins, implicated in the hyperproliferation of VSMC from SHR. To test this, the effects of N-acetylcysteine (NAC), a scavenger of O_2_^-^; PP2, an inhibitor of c-Src; AG1295, an inhibitor of PDGF-R and AG1478, an inhibitor of EGF-R on the enhanced expression of cyclin D1 and cdk4 was examined. Results shown in “Fig 5”, indicate that the enhanced expression of cyclin D1 (A and C) and cdk4 (B and D) in VSMC from SHR were significantly attenuated by NAC (A and B) and PP2 (C and D) towards control levels whereas PP3, an inactive analog of PP2 did not affect the expression of cyclin D1 and cdk4 in VSMC from SHR and WKY rats. On the other hand, PP2 also decreased the expression of both cyclin D1 and cdk4 in VSMC from WKY rats whereas NAC was ineffective. In addition, both AG1295 and AG1478, inhibitors of PDGF-R and EGF-R respectively, attenuated the enhanced expression of cyclin D1 and cdk4 in VSMC from SHR (“Fig 6A, 6B, 6C and 6D”) without affecting the expression in WKY rats.

## 4. Discussion

Hypertension is associated with vascular remodeling which is attributed to the hyperproliferation and hypertrophy of VSMC. Earlier studies showed that NPR-C activation exerts antiproliferative effects in several cell types including vascular smooth muscle cells [17, 26–30]. Hashim et.al have shown that small peptide fragments of cytoplasmic domain of NPR-C with Gi activator sequences as well as C-ANP_4-23_ attenuate vasoactive peptide-induced hyperproliferation of VSMC through Giα/MAPK/P13K/AKT signaling pathways [17]. In addition, C-ANP_4-23_ has also been reported to attenuate the hyperproliferation of VSMC from SHR through the inhibition of enhanced expression of cell cycle proteins, cyclin D1/cdk2/cd4 (G1-S phase) and MAP kinase and Giα protein signalling [19]. Furthermore, a recent Genome- wide association study (GWAS) has shown the presence of two independent BP-related signals within NPR-C gene (NPR3 locus) and that C-ANP_4-23_ and C type natriuretic peptide (CNP) that interact with NPR-C inhibit the hyperproliferation of human VSMC induced by BP elevating allele located on the NPR3 locus [31]. However, in the present study, we demonstrate for the first time that the inhibition of the enhanced oxidative stress, enhanced activation of c-Src and growth factor receptor activation by C-ANP_4-23_ attenuates the overexpression of cyclin D1 and cdk4 in VSMC from SHR and result in the attenuation of hyperproliferation.

We demonstrate that C-ANP_4-23_ treatment decreased high BP in SHR further confirms our earlier study showing that C-ANP_4–23_ attenuated the development of BP in SHR which was associated with inhibition of enhanced expression of Giα proteins and nitroxidative stress [20]. Furthermore, Caniffi et.al also showed that CNP through NPR-C reduced the BP in both SHR and WKY rats, however, the hypotensive effect was more pronounced in SHR compared to WKY rats [32]. The role of NPR-C in the reduction of BP was also shown in NPR-C [NPR3] global knockout mice [33]. However, there have been no studies yet to delineate the role of NPR-C in the attenuation of BP in hypertensive patients.

Oxidative stress is now widely recognized as being a critical player in the pathogenesis of cardiovascular disease including hypertension [24, 25, 34]. The enhanced oxidative stress was shown to be due to the increased production of O_2_^-,^ NADPH oxidase activity and enhanced expression of different subunits of NADPH oxidase [11, 14, 35, 36]. In addition, H_2_O_2_, which increases oxidative stress, also enhances the proliferation of VSMC [37]. However, in this study, we demonstrate that the overexpression of cell cycle proteins cyclin D1 and cdk4 in VSMC from SHR is attributed to the enhanced oxidative stress because the treatment of these cells with NAC, a scavenger of O_2_^-^ attenuated the enhanced expression of these proteins. In addition, the fact that in vivo C-ANP_4-23_ treatment of SHR attenuated the enhanced levels of O_2_^-^ production, NADPH oxidase activity as well as NADPH oxidase subunits p47^phox^ Nox4, Nox1 and Nox2 in VSMC further suggests that C-ANP_4-23-_induced decreased expression of cell cycle proteins is mediated through its ability to decrease oxidative stress which results in the attenuation of hyperproliferation of VSMC from SHR.

The involvement of Giα proteins in the regulation of cell proliferation has been shown [38, 39]. By using pertussis toxin that inactivates Giα proteins and siRNA of Giα proteins, we earlier showed the implication of enhanced expression of Giα proteins in hyperproliferation of VSMC from SHR [14]. In addition, the role of Giα proteins in the enhanced expression of cell cycle proteins and hyperproliferation of VSMC from SHR has also been reported [14, 19]. Furthermore, the role of enhanced oxidative stress in the overexpression of Giα proteins in VSMC from SHR has also been shown [24]. In addition, C-ANP_4-23_ was shown to elicit antiproliferative effect in VSMC from SHR through the inhibition of enhanced expression of Giα proteins [19]. Taken together, it may be suggested that the inhibition of enhanced oxidative stress by C-ANP_4-23_ attenuates the overexpression of Giα proteins which by decreasing the enhanced levels of cell cycle proteins results in the attenuation of hyperproliferation of VSMC from SHR.

Earlier studies have demonstrated the role of non-receptor tyrosine kinase c-Src, a downstream signaling molecule of oxidative stress [11, 25] in the hyperproliferation of VSMC from SHR [11]. Here, we demonstrate for the first time the role of c-Src in enhanced expression of cell cycle proteins cyclin D1 and cdk4 because inhibitor of c-Src, PP2, attenuated the overexpression of these proteins. In addition, we also showed that in vivo treatment of SHR with C-ANP_4-23_ that exerted antiproliferative effect, also attenuated the enhanced activation of c-Src in VSMC from SHR. Taken together, it may be suggested that the antiproliferative effect of NPR-C activation by in vivo treatment with C-ANP_4-23_ may be mediated through its ability to inhibit the enhanced activation of c-Src and resultant inhibition of the overexpression of cell cycle proteins.

Several growth factor receptors are expressed in VSMC and their activation has been shown to induce cell proliferation [40, 41]. We also showed earlier that the enhanced activation of growth factor receptors in VSMC from SHR compared to VSMC from WKY rats contributes to the hyperproliferation of cells [42] and that growth factor receptor activation is the downstream signaling of c-Src because PP2; an inhibitor of c-Src attenuated the activation of growth factor receptors in VSMC from SHR [25]. In the present study, we show for the first time that in vivo treatment of SHR with C-ANP_4-23_ also attenuated the enhanced phosphorylation of EGF-R, PDGF-R and IGF-R in VSMC from SHR. We also report that the inhibitors of PDGF-R and EGF-R attenuated the overexpression of cyclin D1 and cdk4 in VSMC from SHR and suggest the role of EGF-R and PDGF-R in the enhanced expression of these proteins. Taken together, it may be suggested that the inhibition of enhanced activation of growth factor receptors contribute to the antiproliferative effect of C-ANP_4-23_ through the attenuation of overexpression of cell cycle proteins.

**Fig 7:**
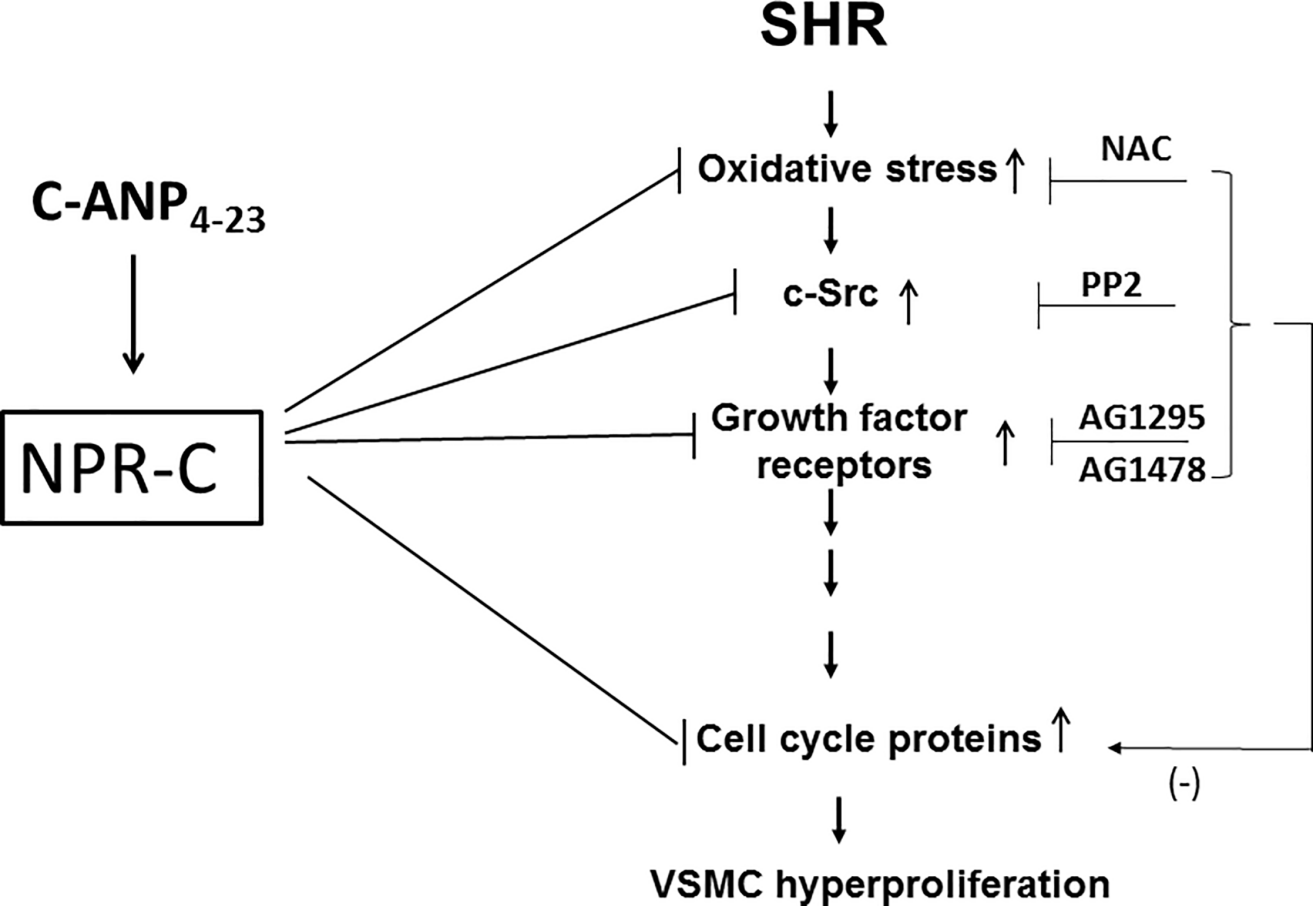
Schematic diagram summarizing the possible mechanisms by which *in vivo* treatment of SHR with C-ANP_4-23_ attenuates the enhanced expression of cell cycle proteins and hyperproliferation of VSMC from SHR.

In conclusion, we demonstrate the implication of enhanced oxidative stress, c-Src and growth factor receptor activation in the overexpression of cell cycle proteins in VSMC from SHR. In addition, we provide the first evidence that the in vivo treatment of SHR with C-ANP_4-23_ through the inhibition of enhanced oxidative stress, c-Src and growth factor receptor activation, attenuates the overexpression of cell cycle proteins that result in the attenuation of hyperproliferation of VSMC from SHR (“Fig 7”). From these studies, it can be suggested that C-ANP_4-23_ could be used as a therapeutic agent in the treatment of vascular complications associated with hypertension, atherosclerosis and restenosis.

## Abbreviations

C-ANP_4-23_: a natriuretic peptide receptor-C (NPR-C) agonist
EGF-R: epidermal growth factor receptor
IGF-R: insulin-like growth factor receptor
NAC: N-acetyl-L-cysteine
NADPH: nicotinamide adenine dinucleotide phosphate
PDGF-R: platelet-derived growth factor receptors
WKY rats: Wistar Kyoto rats
SHR: spontaneously hypertensive rats
VSMC: Vascular smooth muscle cells
